# General versus sports-specific injury prevention programs in athletes: A systematic review on the effect on injury rates

**DOI:** 10.1371/journal.pone.0205635

**Published:** 2018-10-19

**Authors:** Hendrik Mugele, Ashley Plummer, Kathrin Steffen, Josefine Stoll, Frank Mayer, Juliane Müller

**Affiliations:** 1 Department of Sport and Health Sciences, Clinical Exercise Science, University of Potsdam, Potsdam, Germany; 2 Oslo Sports Trauma Research Center, Oslo, Norway; 3 Department of Sport and Health Sciences, University Outpatient Clinic, University of Potsdam, Potsdam, Germany; 4 Department of Computer Science – Therapy Science: Exercise Science and Applied Biomechanics, Trier University of Applied Science, Trier, Germany; Universite de Nantes, FRANCE

## Abstract

**Introduction:**

Annually, 2 million sports-related injuries are reported in Germany of which athletes contribute to a large proportion. Multiple sport injury prevention programs designed to decrease acute and overuse injuries in athletes have been proven effective. Yet, the programs’ components, general or sports-specific, that led to these positive effects are uncertain. Despite not knowing about the superiority of sports-specific injury prevention programs, coaches and athletes alike prefer more specialized rather than generalized exercise programs. Therefore, this systematic review aimed to present the available evidence on how general and sports-specific prevention programs affect injury rates in athletes.

**Methods:**

PubMed and Web of Science were electronically searched throughout April 2018. The inclusion criteria were publication dates Jan 2006–Dec 2017, athletes (11–45 years), exercise-based injury prevention programs and injury incidence. The methodological quality was assessed with the Cochrane Collaboration assessment tools.

**Results:**

Of the initial 6619 findings, 15 studies met the inclusion criteria. In addition, 13 studies were added from reference lists and external sources making a total of 28 studies. Of which, one used sports-specific, seven general and 20 mixed prevention strategies. Twenty-four studies revealed reduced injury rates. Of the four ineffective programs, one was general and three mixed.

**Conclusion:**

The general and mixed programs positively affect injury rates. Sports-specific programs are uninvestigated and despite wide discussion regarding the definition, no consensus was reached. Defining such terminology and investigating the true effectiveness of such IPPs is a potential avenue for future research.

## Introduction

The preventive properties of physical activity to numerous diseases and health-related risk factors are well documented [1,2]. However, personal grief, impairing consequences and high costs make sports-related injuries an unpleasant side effect of participation [3]. Sports injuries represent the second most common type of accident in Germany with 2 million reported cases a year, i.e. 6% of all people participating in sports [4]. Popular team sports, such as soccer, handball, volleyball and basketball, contribute towards almost two thirds of all sporting injuries in Germany [5] with similar trends observed on a global scale [3,6,7]. Within competitive sport, the most frequent injuries are ligament [3,5,6,8,9] and muscle strains [7,8] to the lower extremities both with or without player contact [5,6,8,9]. With these problems highly evident, understanding and managing injury risk is becoming more necessary [10] and is being addressed through the use of injury prevention programs (IPP).

Van Mechelen’s 4-step model [11] for designing an IPP theorizes that incidence rates and mechanisms of injury must be known beforehand. Once achieved, reassessment of incidence is recommended to evaluate the effectiveness of the prevention program on injury risk. With this knowledge, the most appropriate prevention program that results in lowering the risk among the identified high-risk groups can be incorporated. A more recent framework proposed important additional steps that were not originally considered in the 4-step model, that is acknowledgment of the real world challenges that derive from IPP implementation [12]. The Translating Research into Injury Prevention Practice (TRIPP) model theorizes sport bodies will be unwilling to implement sport safety policies until they are accepted by the coaches and athletes [12]. Providing evidence of a given programs’ efficacy alone often fails to positively affect the level of compliance to a program [12]. Coaches are more concerned with maintaining practice that is specific to the nature of the sport (e.g. replicating the movement patterns frequently used in the sport itself) without compromising the athletes’ health and/or performance [12]. For instance, Cumps et al. (2007) found that using sport-specific items was crucial for compliance and implementation of an IPP in basketball players [13]. The definition of specificity of exercise components varies considerably in the literature. The majority of studies term their IPPs as sports-specific [14,15] when they actually include components that develop general physical abilities, e.g. balance, core stability or power. The FIFA 11+ [16], Sportsmetrics [17] and the PEP Program [18] are programs used in team sports to reduce sports-related injuries, whereas other programs are available that target specific injury types [19,20]. These are all examples of IPPs that comprise of both general and sports-specific exercises and have been shown to efficaciously reduce the rates of ankle, knee and lower limb injuries [13,16,21–24]. Yet, there is no consensus about the “best” exercise or mixture of exercises to prevent sport injuries in athletes. Consequently, the ratio of general and sports-specific components in an efficacious IPP is uncertain. Despite not knowing about the superiority of sports-specific IPPs, coaches and athletes alike prefer more specialized rather than generalized exercise programs. Does the available evidence justify the common coaches’ consensus that strictly sports-specific IPPs are the most effective in reducing injury rates while enhancing performance [12,13,25,26]? Or are multifaceted or generalized approaches adequate so that sport therapists and/or the coaching staff do not necessarily have to implement IPPs specific to their athlete’s? For instance, could a coach use the same IPP on players from soccer and volleyball or is implementation of a soccer-specific and volleyball-specific IPP preferred.

Therefore, the current systematic review aims to examine the efficacy of sports-specific and general prevention programs in preventing sport injuries in athletes.

## Methods

In accordance with the Preferred Reporting Items for Systematic Reviews and Meta-Analyses [27], a systematic search of the literature was conducted. Neither a review protocol or registration information including registration number exists.

The electronic databases of PubMed and Web of Science were systematically searched for relevant articles between Jan 2006 until Dec 2017. English-language publications in human populations with restriction to interventional study designs were included, i.e. randomized controlled trails (RCT) and controlled trails (CT). The search terms used were “*athletes”* AND “*injury prevention”* OR “*exercise program*”. Two authors (HM; AP) performed the literature search independently with disagreements resolved by consensus and if necessary, with further consultation from a third author (JM). The search process consisted of removing duplicates, screening titles, abstracts and eligible full texts. Additionally, reference lists of excluded systematic reviews, meta-analyses and reviews were manually checked for studies of relevance.

### Eligibility criteria

**Fig 1:**
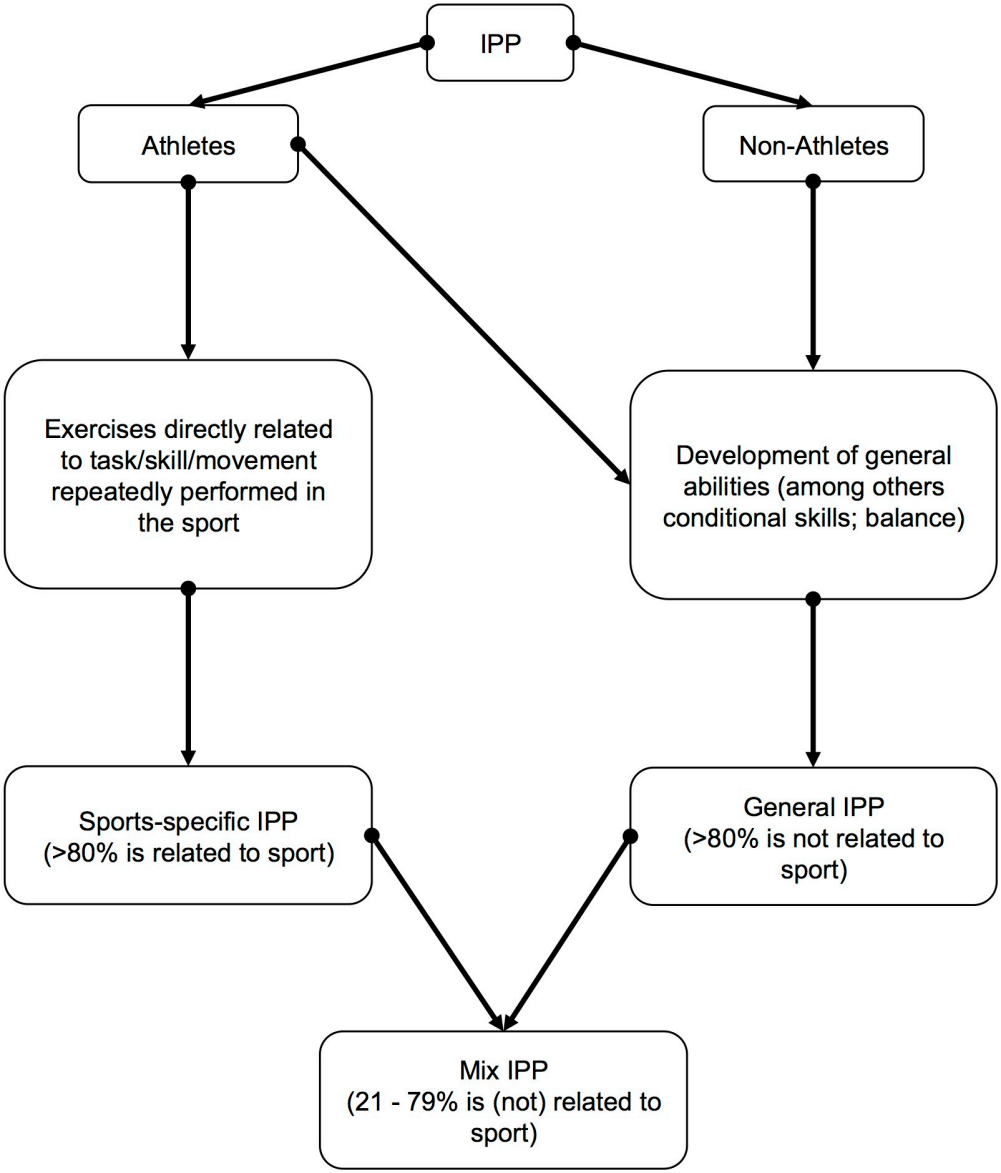
Author’s definition of general, sports-specific and mixed injury prevention programs (IPP).

Athletes (11–45 years) from various sports disciplines had to participate in an exercise-based IPP or kept their usual training routine or standardized protocol. General and sports-specific IPPs were interpreted by the authors (Fig 1) and were subsequently categorized based on this assumption. More precisely, the nature of individual exercises of the IPP was to be determined. Independent of each other, the two authors (HM; AP) categorized the exercises into general and sports-specific according to whether those do replicate the tasks, skills or movements repeatedly performed in that particular sport with disagreements resolved by further consultation. Passive IPPs, among others based on stretching, were considered irrelevant and thus were excluded. Furthermore, studies had to state principal summary measures, such as injury rates, risks and severities.

### Data extraction

The following data were extracted from each eligible full text: the first author’s last name, publication year, study design, country of origin, follow-up period, study duration, level, type of sport, exposure data, subject information (sample size, dropout rate, sex and age), intervention (name, description, type, dose, frequency, compliance, effects and categorization according to authors’ definition) and injury data (locations, types, mechanisms and rates). Throughout the studies injury rates were expressed as 1000 hours of exposure [13,21–24,28], 1000 player hours [16,29–32], per 1000 hours [33,34], 1000 athletes-exposure [35–42], per sports hour [43] 100 player seasons [44], time until first incident and one season prevalence [19,45]. Injury risks were stated as odds ratios (OR) [19,31,37,46], relative risk (rr) [13,23,29,31,38,42,47], rate ratio (RR) [16,24,28,30,33,34,40,41], adjusted rate ratio (aRR) [44], adjusted and unadjusted incidence rate ratio (IRR) [22,32,36] and hazard ratio (HR) [21,48].

### Risk of bias assessment

The Cochrane Collaborations’ risk of bias assessment tool [49] was used to evaluate the internal validity of the included RCTs. Independently, the two authors (HM; AP) examined the studies of interest for the following sources of bias: selection (sequence generation and allocation concealment), performance (blinding of participants/personnel), detection (blinding outcome assessors), attrition (incomplete outcome data), reporting (selective reporting) and other potential bias (e.g. recall bias). Additionally, included CTs were assessed with the Cochrane risk of bias in non-randomized studies (NOS)–of Interventions (ROBINS-I) assessment tool [50]. This tool assesses the risk within specific domains, such as bias due to confounders, selection, intervention, missing data and measurement of outcomes.

## Results

**Fig 2:**
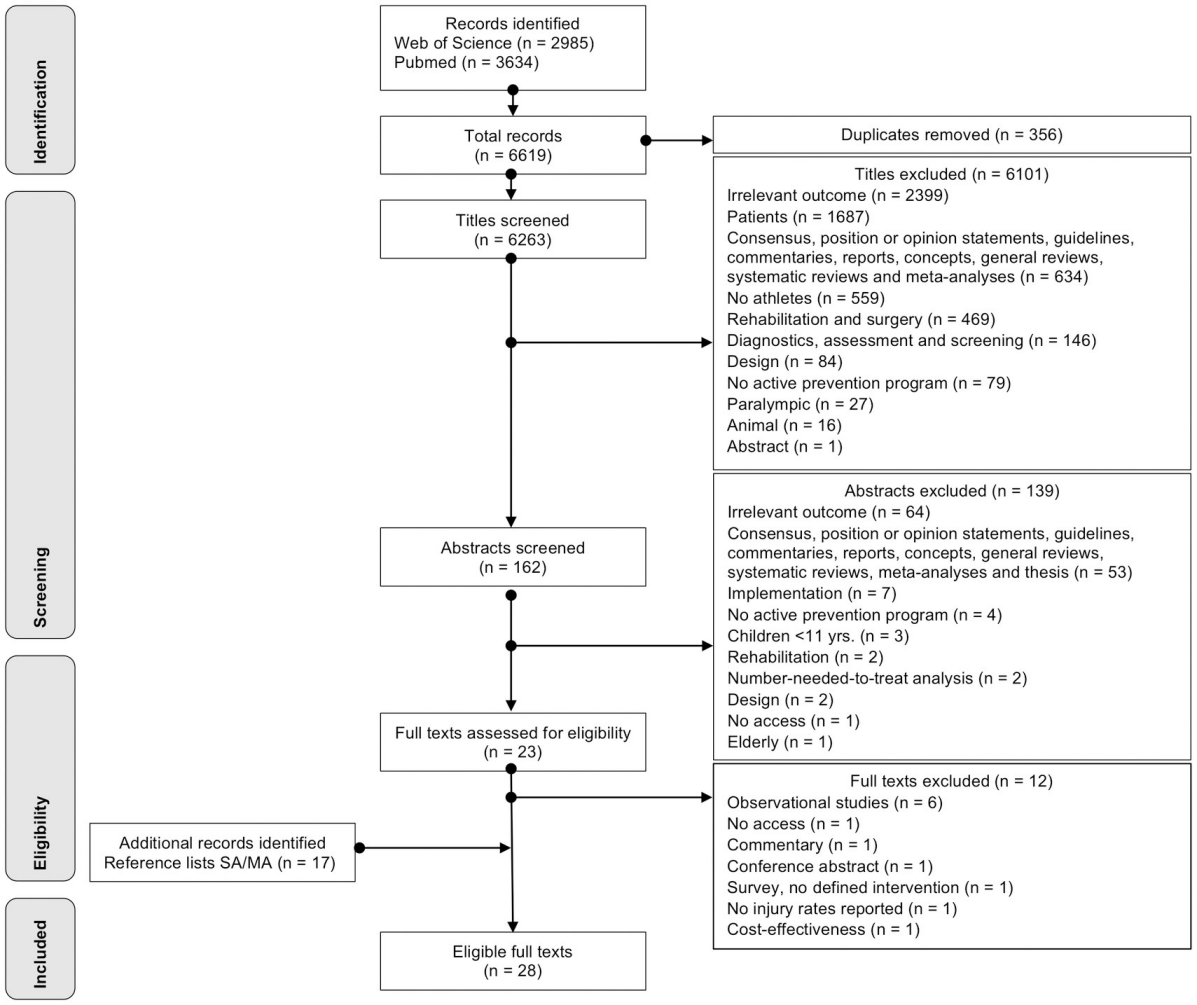
Flowchart for screening and selection of studies according to PRISMA.

The initial search strategy identified 6619 studies. After screening titles and abstracts, a total of 6596 were excluded because they were either duplicates, reports, general review articles, current concepts, commentaries, systematic reviews, meta-analyses, off-topic or failed to match the inclusion criteria. After the assessment of the remaining 23 full text articles, a further eight studies were excluded. Additional evidence was retrieved by reference lists of previously excluded suitable reviews, which unearthed a further 13 eligible articles. Consequently, 28 articles [13,16,19,21–24,28–48] were available for final evaluation (Fig 2).

### Study characteristics

Detailed information about study characteristics and results as well as interventions can be found in the supplementary material (S1, S2 and S3 Tables).

The majority of studies used team sport athletes from soccer [16,24,28,30–36,38,40–44,48], basketball [13,21,29,36,38,46], handball [19,45], floorball [22], Australian football [47] and mixed sports (i.e. athletes from more than three different sports) [23,39]. Seventeen studies used amateur [16,21,23,24,28–33,36,38–41,43,48], 10 elite [13,19,20,22,34,35,37,42,45,46] and two studies combined amateur and elite athletes [44,47]. The average sample size was 479 athletes (range: 20–2479; NTotal = 13411) for intervention and 468 athletes (range: 20–2085; NTotal = 13117) for control groups. In both groups, athletes’ age ranged from 13–45 years.

Of all IPPs, three consisted of a single exercise, which was implemented into the athletes’ training routine [31,44,47]. Another two IPPs consisted of a home-based program [23,32], whereas 21 were on-field [13,16,19,21,22,24,28–30,32–38,40,41,43,45,48] warm-up programs with an emphasis on either running, plyometrics, agilities, strengthening, balance, flexibility, proper landing technique and awareness of injury mechanisms. Furthermore, three other studies incorporated a whole additional training program [39,42,46]. In addition to usual training routine, the IPPs’ temporal characteristics averaged out to 1–6 weekly sessions for 5–90 min. over four weeks up to four years. The studies were categorized into seven general [23,24,31,42,44,47,48], 20 mixed [16,19,21,22,28–30,32–41,43,45,46] and one sports-specific [13]. Eleven studies [15,16,28,29,32–34,37,40,41,43] recorded all acute, severe and overuse injuries sustained during training and match exposures, whereas four studies [21,22,36] focused on preventing lower extremity injuries only. Other articles documented injury rates on the knee [13,23,38,42,46], hamstring [31,44,47], ankle [13,23,38,42,46], shoulder [19,45] and groin [48].

### Injury rates

The results of the IPPs’ effectiveness are presented according to author’s definition and are categorized into three sections: 1) general, 2) mixed and 3) sports-specific programs.

#### General IPPs

All the studies comprising a general IPP trained the lower extremity (ankle [23,42]; knee [24]; groin [48]; hamstring [31,44,47]). The IPPs with 1–10 exercises took about 10–30 min. to complete and were applied 1–4 times per week. All studies revealed a significant effect on injury incidence following the interventions apart from the groin warm-up program (HR 0.69 (95% CI 0.40–1.19)) [48].

The Nordic hamstring exercise yielded in a significant reduction in overall (aRR 0.293 (95% CI 0.150–0.572)), new (aRR 0.410 (95% CI 0.180–0.933)) and recurrent hamstring injuries (aRR 0.137 (95% CI 0.037–0.509)) between intervention and control group [44]. Moreover, a significant hamstring risk reduction, but not in injury severity, was found during the follow-up in one study [31] (OR 0.282 (95% CI 0.110–0.721); p = 0.005), whilst another [47] only found a protective effect of the Nordic hamstring protocol when athletes comply with at least the first two sessions (rr 0.3, 95% CI 0.1–1.4; p = 0.098).

After implementation of a neuromuscular training program, a significant 64% reduction of overall anterior cruciate ligament (ACL) injuries (RR 0.36 (95% CI 0.15–0.85)) in the intervention group was reported [24]. Additionally, the adjusted subgroup analysis revealed an 83% decrease in total (RR 0.17 (95% CI 0.05–0.57)) as well as noncontact ACL (RR 0.26 (95% CI 0.07–0.99)), acute (RR 0.53 (95% CI 0.30–0.94)) and severe knee injuries (RR 0.18 (95% CI 0.07–0.45)) in the intervention group.

The rate of ankle injuries was significantly reduced following a proprioceptive training compared to control, strength training and/or orthosis (rr 0.13 (95% CI 0.003–0.93); p = 0.02) [42].

#### General IPPs and previously injured athletes

A balance board warm-up program significantly reduced the recurrence rate of ankle sprains (rr 0.63 (95% CI 0.45–0.88)), totaling in a risk reduction of 35% and less severe injuries (rr 0.53 (95% CI 0.32–0.88)) in the intervention group [23]. Noticeably, untreated ankles benefited more from the IPP compared to their control group counterparts.

#### Mixed IPPs

Of the 20 studies comprising mixed IPPs, nine trained the lower [16,21,22,29,30,35,38,39,46], two the upper [19,45] and eight both lower and upper extremities [28,32–34,37,40,41,43]. The IPPs included 3–36 drills, which took 5–30 min. and were applied on 1–6 sessions per week. Three studies failed to find an effect on injury rates to the lower and upper extremities [28,33,43].

The Oslo Sports Trauma Research Center Shoulder Injury Prevention Warm-up Program significantly reduced the prevalence of shoulder problems by 28% (OR 0.72 (95% CI 0.52–0.98)) but not in substantial shoulder problems (OR 0.78 (95% CI 0.53–1.16)) in the dominant arm during one season in the intervention compared with the control group [19]. Another study focusing on shoulder complaints in handball players found reduced problems in the intervention group (pre- 34% and post- 11%) [45]. The FIFA 11+ showed a significant effect on impairments to the trunk (OR 0.09 (95% CI 0.11–0.72)) in the intervention compared to control group [37].

A neuromuscular warm-up [36] and balance training program [38] reduced ankle sprains by 66% and 38%, respectively. Moreover, a multistation proprioceptive training decreased the odds of receiving an injury to the ankle (OR 0.355 (95% CI 0.151–0.835); p = 0.018) [46]. However, the FIFA 11+ (RR 0.65 (95% CI 0.48–0.87)) [41] and neuromuscular prevention program [32] (IRR 0.5 (0.24 to 1.04)) did not change the rate of ankle injuries. Furthermore, ankle sprains were reduced by the balance-training program (rr 0.71 (95% CI 0.45–1.13)); however, did not differ significantly between both groups but were more frequently in the control group (53.9% (95% CI 45.3–62.3)) [29]. Similar results were found using the FIFA 11+ [16,37] and a neuromuscular training program [22] (IRR 0.28 (95% CI 0.12–0.67)). The FIFA 11+ [40,41], PEP program [35], HarmoKnee [30] and a neuromuscular-training program [36] all significantly reduced the incidence of injury to the knee. Although not significant, a reduction in knee ligament injury (IRR 0.49 (95% CI 0.18–1.31)) using another neuromuscular training program was found [22]. For instance, the HarmoKnee [30] resulted in an overall 77% (RR 0.23 (95% CI 0.04–0.83)) reduction of knee injuries. Moreover, the intervention decreased the incidence of noncontact knee injuries by 90% (RR 0.10 (95% CI 0.00–0.70). Similar results were found using the FIFA 11+ with significantly fewer overall knee (RR 0.42 (95% CI 0.29–0.61); p < 0.001), match and training (RR 0.59 (95% CI 0.52–0.68); p < 0.001 and RR 0.46 (95% CI 0.38–0.57); p < 0.001, respectively) as well as noncontact ACL injuries (RR 0.25 (95% CI 0.06–1.15); p = 0.049) compared to a control group [40]. The same research group found a reduced incidence in overall ACL injuries (RR 0.236 (95% CI 0.193–0.93); p < 0.001) [41]. Noncontact ACL injuries sustained with history of ACL injury were significantly lower following the PEP program [35]. The Sportsmetrics also showed a preventive effect for noncontact ACL injuries with an incidence rate of 0.03 (per 1000 athlete-exposures) (p = 0.03) [39].

Alongside a reduction of 41% in overall soccer injuries (RR 0.59 (95% CI 0.40–0.86); p = 0.006), injuries to the lower extremities were significantly reduced by the FIFA 11+ by 41% (RR 0.52 (95% CI 0.34–0.82); p = 0.004) with less severe injuries (RR 0.52 (0.23–1.21); p = 0.037) in the intervention group [34]. The FIFA 11+ [41] resulted in significantly lower rates of hamstring injuries (RR 0.37 (95% CI 0.21–0.63); p < 0.001) in the intervention group.

#### Sports-specific IPPs

Only one study comprised a sports-specific IPP targeting the lower extremities (i.e. ankle [13]). During one season, three sessions of 5–10 min. with 12 exercises were applied per week.

The basketball-specific balance training, taking total exposure time and time spent on basketball activities into account, showed significant lower relative risk of sustaining lateral ankle sprains in the intervention compared with the control group (rr 0.34 (95% CI 0.12–0.96)) and (rr 0.30 (95% CI 0.11–0.84)), respectively [13]. Furthermore, new and re-injuries were not significantly affected between groups regardless of the exposure.

### Risk of bias assessment

**Fig 3:**
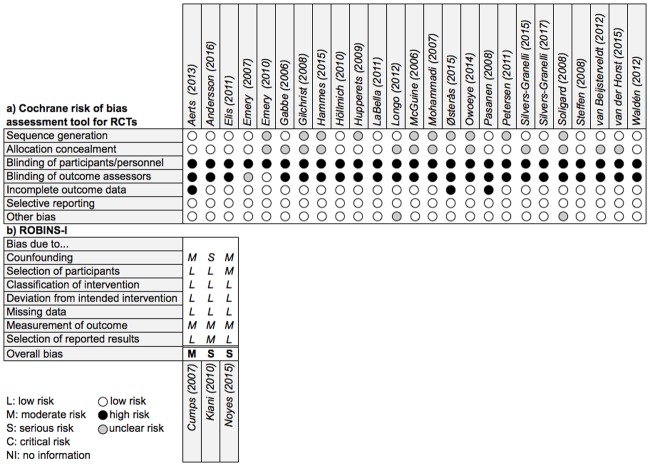
Risk of bias assessment of included trials a) RCTs and b) NOS.

The results of the methodological quality assessment across all included studies separated by RCTs and NOS are summarized in Fig 3a) and 3b).

#### RCT

An appropriate procedure for a randomly generated sequence was described in 15 RCTs [19,21,22,24,28,29,31,36,37,40,41,43,46–48] whilst nine [16,23,33–35,38,42,44,45] did not report any method used for randomization. Furthermore, 12 studies [19,21–24,28,29,36,44–46,48] concealed the allocation. Performance bias was found across all trials. Apart from two [29,32], all studies induced detection bias. A high risk of incomplete outcome data was found in three trials [21,22,45]. Two studies [16,37] showed an uncertain risk of bias due to other threats to internal validity.

#### NOS

The studies showed moderate to serious risk. In the confounding domain one study [30] was of serious risk whereas the remaining [13,39] of moderate risk. Selection of participants, intervention and missing data were found on average to be low of bias. One study [39] had moderate problems in participant selection. The outcome measurement and reporting domain showed moderate risk and low risk of bias, respectively. Only one study [30] resulted in moderate risk in the reporting section.

## Discussion

The purpose of the systematic review was to investigate sports-specific versus general IPPs and their effectiveness on injury prevention. The main finding of the research was that the high majority of interventions led to a reduced risk of sports-related injuries. Only four studies did not reduce injury rates compared to the control group (one general [48], three mixed [28,33,43] IPP).

### Influence of IPPs

The interpretation of what constitutes a sports-specific IPP provides the foundation of the research question, and within the literature, this varies considerably. Studies that termed their programs as sports-specific contrasted to the author’s definition [14]. For instance, programs developed by the FIFA Medical Assessment and Research Center (F-MARC), e.g. FIFA 11+, are widely accepted as soccer-specific [14] but yet have been proven efficacious beyond soccer, such as in basketball [37]. It seems more applicable to refer to such regimes as multifaceted (mixed IPPs), as they address many factors potentially related to the risk of injury [16]. In spite of the frequently claimed need for sports-specific programs by coaches, those, by the authors’ definition, are yet to be vigorously assessed. The aforementioned missing consensus about the design of a sports-specific IPP could be one possible reason for the lack of implementation of those programs. Nevertheless, it was assumed that the proper execution of an exercise is more important for the prevention effect than the selection of an exercise that can be deemed specific [15].

The majority of evidence focused on multi interventional and/or general approaches which is in agreement with the literature that suggested by implementing plyometric, balance, resistance, agility and/or flexibility exercises, multifaceted IPPs efficaciously prevent hamstring and ACL injuries by altering various risk factors [51]. No study referred to the existence of the “best” exercise or mixture of exercises to prevent injuries in athletes. The Nordic hamstring exercise efficaciously reduced the number of hamstring injuries and various risk factors in athletes [20,44,51–54] and therefore, this may be an example for one “best” exercise to prevent muscle strains in hamstrings across a wide range of sports disciplines. Future investigations, ideally RCTs, need to address individual exercises from previously proven effective IPPs in order to determine its efficacy for reducing injury rates. As the major reason for both coaches and athletes to not implement evidence-based IPPs into their usual training routine is the loss of training time, IPPs must be shorter (i.e. more efficient) and therefore, ineffective components with no relevance for preventing sport injuries in athletes need to be identified and removed. Additionally, future trials need to be wary of the inherent problems that derive from methods, such as self-reporting and injury documentation as well as the implementation, execution and compliance to an IPP itself. This; in turn, requires time and labor as well as resources. Providing that these assumptions are met, evidence may result in a collection of “best” prevention exercises for a variety of sport injuries and thus IPPs can be designed to ensure even more efficacious sport injury prevention. Identifying those exercises may be more important than focusing on the aspect of specificity, i.e. general or sports-specific; however, this alone is not enough for coaches and/or athletes to compliantly implement exercise components.

Also, a possible aspect to consider is movement specificity. For instance, team sports, such as soccer, basketball, handball and rugby, share the same fundamental movement pattern typical for injury risk situations, i.e. cutting maneuvers with multiple acceleration and deceleration of the athletes’ body weight as well as jumping [55,56]. Furthermore, lower competition level is associated with higher injury incidence [57]. A possible explanation for this observation could be too little exposure to the movement-specific exercises in their training compared to higher-level athletes. In other words, each athlete’s training needs to mimic those of competition in order to compete efficiently and effectively. Harre (1982) described this as the “Principle of Specificity” [58]. Therefore, it could be reasonably assumed that the emphasis of programs should be focused towards more movement-specific components, as this would ensure optimal performance under the aspect of low incidences of injuries in exceptional positions.

Mixed Programs to prevent injuries were largely successful. It was shown that using a wide array of exercise that pertain to both sports-specific and general movements were effective in reducing overall injury rates of the sport. However, the necessity of combining these components in an IPP remains unclear, although, with the goal of reducing injury rates in athletes and time loss due to injury the evidence would seem to strongly indicate this [59]. Another aspect is the timing of the IPP implementation, as incidence rates are shown to be greater in the first four weeks of season [60]. Therefore, it could be reasonably postulated that maintaining a certain level of conditioning during off-season is integral, as it exposes athletes to continued bouts of specific movements before a deconditioning effect occurs.

Conversely, the overloading effect caused by repetitive sports-specific movements represents another potential confounder as illustrated by one study [37] whom after using the FIFA 11+, reported more overuse injuries when compared to control. Since the prevalence is directly linked to volume and frequency [61], performing specific jumping movements as part of a neuromuscular training would add to the already high loading pattern during the season.

Heterogeneous characteristics across the included studies make a comparison of different exercise components practically impossible. Differences in IPPs frequency, duration and timing as well as form of implementation, supervision and control varied largely across studies. A dearth of supervision might be a possible explanation for a low compliance to home-based but also on-field interventions as adherence is required from athletes and coaches alike [62]. Furthermore, compliance and incidence rates were shown to have a potential inverse relationship [62]. For instance, the ‘11+ Kids’ program [59] found a decreased incidence of overall, severe and lower extremity injuries when compliance was adequately high. A great variety of methods were used to calculate incidence rates and exposure data. Additionally, studies emphasized pre defined anatomical sites (i.e. knee [24,30,35,39], hamstring [31,44,47], ankle [13,23,38,42,46], shoulder [19,45] and groin [48]) or lower/upper extremity further divided into overall, training and match injuries [16,21,22,28,29,32–34,36,37,40,41,43], which makes comparisons problematic. Moreover, sex, age and athletic abilities of the participants restrict the value of the research when making practical recommendations. Consistent with other authors [24,28,44,48,63], low compliance rates, inadequate sample sizes, self-reporting and selection bias represent prominent problems across injury prevention studies. Self-reporting of injuries either by athletes, coaches or other personnel can easily lead to injury misclassifications and exposure data due to recall bias [15]. Additionally, a poor follow-up does not allow conclusions to be drawn on long-term effects of an IPP, which reduces its practical application. Moreover, cluster randomization can be a double-edged sword. On the one hand, it has the opportunity to eliminate contamination bias [24] but conversely, a high dropout rate within an individual cluster results in a detrimental loss of data and induce bias [64].

### Recommendations

The findings of the systematic literature search suggest that multifaceted and general IPPs have great potential in reducing the risk of injury among athletes. This is in agreement with other systematic reviews previously published [65–67]. Additionally, they can be easily implemented into the normal training routine. It was shown that one efficient strategy to implement an IPP is to use the warm-up part (10–20 min) during each training session [59,67]. Furthermore, IPPs should be applied throughout the entire year [65] and emphasis on proper technique, not complexity of exercises [15].

### Risk of bias assessment

Overall reporting throughout the included RCTs was adequate. An inherent yet unavoidable issue is that the blinding of participants is not possible in exercise interventions; therefore, all trials showed high risk of performance/detection bias. Nevertheless, the overall quality could have been underestimated by secretive reporting. Therefore, transparent reporting across all studies is mandatory and should ideally follow consensus statements such as CONSORT [27].

The most problems across NOS studies were found in the domains of confounding and outcome measurement. This finding is consistent with the included RCTs. The general reporting in the studies was not adequate; therefore, guidelines like the STROBE [68] were developed and are recommended to improve transparency.

### Study limitations

The initial search strategy had a lack of robustness with only two databases been searched. It must also be noted many articles were found during the manual scanning process of reference lists from related systematic reviews. This raises further concern over the used search strategy and keyword combinations. It may be that the terms were too specific and therefore, could have resulted in a limited number of identified articles. To further advance the search strategy, additional more sports-specific search terms, i.e. injury sites and/or sports disciplines, would have been beneficial. In turn, this might have ameliorated distinct conclusions by allowing comparisons between exercise components in regard to their injury preventing effect in a matched population, i.e. in a particular sport disciplines (e.g. soccer), injuries to a specific side of the body (e.g. ACL rupture), equal age group and performance level.

### Future research

To facilitate discussions amongst therapists and coaching staff, it would be of benefit if a consensus regarding a definition of what a general and what a sports-specific IPP could be is reached. Furthermore, to the authors’ knowledge, there are no trials that compare a genuine sports-specific intervention against a general IPP when examining the effects on injury risk. In order to make more specific conclusions, future investigations should try to expand their focus towards different sport disciplines, age groups, competition levels, specific injuries and/or previously injured athletes when it comes to the efficacy of an IPP. In turn, this might help to shorten IPPs by identifying ineffective components or the “best” exercise and/or mixture of exercises for preventing sport injuries in athletes.

### Conclusion

IPPs contribute to a reduction of the risk of sustaining sports injuries in athletes. The current evidence indicates that general or mixed IPPs tend to be more efficacious in preventing sport injuries in athletes. However, it is worth noting that sports-specific IPPs are uninvestigated to date.

## Supporting information

S1 Table Summary of intervention programs used in the included studies (alphabetical order by program). ACL, anterior cruciate ligament; BB, basketball; CPL, compliance; EX, exercise(s); KLIP Program, Knee Ligament Injury Prevention Program; LE, lower extremity; min., minutes; N/D, not described; OSTRC, Oslo Sports Trauma Research Center; PEP Program, Prevent injury and Enhance Performance Program; Pt., part/phase; reps, repetitions; s, seconds; UE, upper extremity; VB, volleyball; wk., week(s); yrs., years.
(DOCX)

S2 Table Summary of characteristics of included studies for the injury rates (alphabetical order by first author). Level**: P, professional/ elite/ highest level; AM, amateur; C, coaches; cS, competitive season; CT, controlled trail; M, match/game; min., minutes; mo., months; N/D, not described; reps, repetitions; S, season; T, training/practice; wk., weeks; yrs., years. Values presented as mean ± standard deviation if not otherwise stated.
(DOCX)

S3 Table Results and conclusion of included studies for the injury rates (alphabetical order by first author). *^B^: Statistically significant difference between intervention group and baseline. ‘’: Statistically significant difference within groups. *: Statistically significant difference between intervention and control group. ^#^: Statistically significant difference compared to all groups. A, athletes; ACL, anterior cruciate ligament; AE, athletes-exposure; aRR, adjusted rate ratio; CI, confidence interval; CG, control group; CO, compliant group; cS, competitive season; CT, controlled trail; d, days; E, exposure; EX, exercise; h, hours; IG, intervention group; INT, intervention team; IR, injury rate; IRR, adjusted incident rate ratio; LE, lower extremity; M, match, game; MD, mean difference; min., minutes; mo., months; N/A, not available; NCO, not compliant group; no., number; OR, odds ratio; pS, pre-season; RR, rate ratio; rr, relative risk; S, season; SD, standard error; T, training, practice; wk., weeks; yrs., years. Values presented as mean ± standard deviation if not otherwise stated.
(DOCX)

S1 PRISMA checklist PRISMA checklist. (PDF)
