# Supplementary material for: General versus sports-specific injury prevention programs in athletes: A systematic review on the effect on injury rates
Source: PLoS One. 2018 Oct 19;13(10):e0205635. doi: 10.1371/journal.pone.0205635 (PMC6195266; doi:10.1371/journal.pone.0205635)
Supplement: S1 Table — ACL, anterior cruciate ligament; BB, basketball; CPL, compliance; EX, exercise(s); KLIP Program, Knee Ligament Injury Prevention Program; LE, lower extremity; min., minutes; N/D, not described; OSTRC, Oslo Sports Trauma Research Center; PEP Program, Prevent injury and Enhance Performance Program; Pt., part/phase; reps, repetitions; s, seconds; UE, upper extremity; VB, volleyball; wk., week(s); yrs., years. (DOCX) [file pone.0205635.s001.docx]

| ***S1 Table****. Summary of intervention programs used in the included studies (alphabetical order by program)* | | | | |
| --- | --- | --- | --- | --- |
| **Program**  **(Year)** | **Intervention Details** | **Dynamic warm-up** | **Plyometrics** | **Balance** |
|  |  | **Strength** | **Agility** | **Flexibility** |
| **Mixed approaches** | | | | |
| *Aerts et al.*  *(2013)^21^* | 5 - 10 min. progressive warm-up program with monthly change of program emphasis (technique, fundamentals and performance).  2 times per week for 3 month.  CPL: 66.7% | None | Pt. 1 wk. 1: 1 jump EX  wk. 2: 1 jump EX  wk. 3: 4 jump EX  wk. 4: 5 jump EX Pt. 2 wk. 1 - 4: 3 jump EX Pt. 3 wk. 1 - 4: 5 jump EX | None |
|  |  | Pt. 1 wk. 1 - 2: 4 EX (LE)  wk. 3: 1 EX (LE)  wk. 4: None Pt. 2 wk. 1 - 4: 2 EX (LE, core) Pt. 3 wk. 1 - 4: None | None | None |
| Eils *et al.*  *(2010)^46^* | 20 min. proprioceptive exercise program  1 time per week for 9 - 18 weeks.  CPL: N/D | None | 1 jump EX | 5 EX |
|  |  | None | None | None |
| *Emery et al.*  *(2007)^29^* | 15 - 20 min. prevention program consisting of a standardized warm-up (10 min.) with additional warm-up component (5 min.). Additionally, home-based balance training program using a wobble board (20 min.) with progression exercises.  5 times per week for 18 weeks.  CPL: 60.3% (home) | Jogging and running EX | None | Sport-specific    Home EX (wobble board) |
|  |  | None | None | Static Dynamic |
| *Emery et al.*  *(2010)^32^* | 15 - min. warm-up routine, including 5 min. of aerobic, static stretching and dynamic stretching components (same as control) in addition to 10 min. of neuromuscular training components (i.e. strength, agility and balance). Additionally, a 15 - min. home-based balance training program was included.  15 min. per training session and study period was 1 year.  CPL: N/R (on-field); < 15% (home) | None | 1 jumping EX | 2 (LE) |
|  |  | 1 (core)  2 (LE) | None | None |
| Continued | | | | |

| Continued | | | | |
| --- | --- | --- | --- | --- |
| **Program**  **(Year)** | **Intervention Details** | **Dynamic warm-up** | **Plyometrics** | **Balance** |
|  |  | **Strength** | **Agility** | **Flexibility** |
| *FIFA 11+  (2012)^37^*  *(2014)*^34^*  *(2014)^#33^*  *(2015)^+41^*  *(2017)^&40^*  *The 11+/F-MARC 11+*  *(2008)^¶16^* | 20 min. three component warm-up program with 3 difficulty stages.  3 - 4 times per week for 9 months. Only the running exercises on match days.*^#+&^ 5 - 6 times per week for one season.¶∞  CPL: 100%; 74%*; 98%^#^; moderate^+^ 73%^&^  CPL: 77%^¶^ | 6 running drills*^¶∞^ | 2 jumping EX*^¶∞^ | 1 EX*^¶∞^ |
|  |  | 3 EX (LE, core)*  4 EX (LE, core)^¶∞^ | 2 drills*^¶∞^ | None*^¶∞^ |
| *The 11/F-MARC 11  (2008)***^28^*  *(2012)^&43^* | 10 – 15 min. warm-up program with 10 evidence-based exercises focusing on core stability, balance, dynamic stabilization and eccentric hamstring strength.  Every practice (15 consecutive sessions) thereafter once a week for 8 months.* Every practice for 4 yrs..^¶^  2 times per week for 20 weeks.^∞^ CPL: 52%*; 73%^&^ | Jogging*^∞^  N/D^¶^ | 2 jumping EX*^¶∞^ | 4 EX (ball, pair)^¶^  4 EX (ball, pair, balance mat)*^∞^ |
|  |  | 2 EX (LE, core)^¶^  3 EX (LE, core)*^∞^ | 1 drill*^¶∞^ | None*^¶∞^ |
| *HarmoKnee*  *(2010)^30^* | 20 - 25 min. knee injury prevention program to increase overall awareness of injury risk and aiming on achieving an improved motion pattern.  2 times per week (preseason) and once (in season) for 9 months. CPL: 94% recalled ≥ 75% | 5 jogging and running EX  6 "muscle activation" EX (LE, hip/groin, lower back) | 4 jump EX | None |
|  |  | 6 EX (LE, core) | None | None |
| *LaBella et al.*  *(2011)^36^* | 20 min. progressive neuromuscular warm-up training before each practice (including strengthening, plyometrics, balance and agility exercises) with abbreviated version before matches for one entire season. Compliance: 80% | Jogging 16 running technique | wk. 1: 7 jumping EX  wk. 2 -3: 8 jumping EX  wk. >4: 7 jumping EX | None |
|  |  | wk. 1: 7 EX (LE, core)  wk. 2: 9 EX (LE, core)  wk. 3: 10 EX (LE, core)  wk. 4+: 9 EX (LE, core) | 3 drills | None |
| McGuine *et al.*  *(2006)^38^* | 10 - min. program consisting of double- and single- leg stance exercises. Progressions were: flat surface to balance board; eyes open to closed and integrating functional sport activities such as throwing, catching and dribbling on 1 leg.  3 - 5 times per week for 1 season  CPL: N/R | None | None | wk. 1: 3 (LE)  wk. 2: 2 (LE)  wk. 3: 4 (LE)  wk. 4+: 3 (LE) |
|  |  | wk. 1 - 2: 1 (LE)  wk. 4 - 5+: 1 (LE) | None | None |
| Continued | | | | |

| Continued | | | | |
| --- | --- | --- | --- | --- |
| **Program**  **(Year)** | **Intervention Details** | **Dynamic warm-up** | **Plyometrics** | **Balance** |
|  |  | **Strength** | **Agility** | **Flexibility** |
| *OSTRC Shoulder Injury Prevention Program   (2016)^19^* | 10 min. warm-up to prevent shoulder injury by improving glenohumeral internal and external rotation, scapular muscle strength, kinetic chain and thoracic mobility. Additional difficulty levels.  3 times per week prior to throwing activities for 7 months.  CPL: 64% - 93% | None | None | None |
|  |  | wk. 1 - 6: 3 EX (core, UE)  wk. 7 - 12: 3 EX (core, UE)  wk. 13-18: 3 EX (core, UE)  wk. >18: EX of choice | None | wk. 1 - 6: 2 EX (UE) wk. 7 - 12: 2 EX (UE) wk. 13 - 18: 2 EX (UE) wk. 18 - 24: 1 EX (UE) wk. >18/24: EX of choice |
| *Østerås et al.*  *(2015)^45^* | 10 - 15 min. shoulder muscle strength program as a prolonged warm-up.  3 times per week for 8 months. COL: 71% | None | None | None |
|  |  | 3 EX (UE, core) | None | None |
| *Pasanen et al.*  *(2008)^22^* | 20 - 30 min. neuromuscular warm-up program to enhance motor skills, body control, activation and preparation of the neuromuscular system for sports specific maneuvers with 3 difficulty levels.  2 - 3 times per week for 6 months.  CPL: 43% - 82% | Jogging  8 running technique  Speed run | 1 of 3 EX | 1 of 3 EX (even surface, balance board, medicine ball) |
|  |  | 1 of 3 EX (LE)  1 of 3 EX (core) | None | Only for impaired players  3 EX (LE, hip, back) |
| *PEP Program*  *(2008)^35^* | 30 min. warm-up program focuses on ACL injury prevention by stretching, strengthening, plyometrics, agilities and avoidance of high-risk positions.  Replacement exercises were given.  3 times per week 12 weeks.  CPL: 71.1% | 3 jogging and running drills | 5 jump EX | None |
|  |  | 3 EX (LE) | 3 drills | 5 EX (LE) |
| *Sportsmetrics*  *(2015)^39^* | 90 – 120 min. progressive neuromuscular training program consisted of a general dynamic warm-up, jump and flexibility training and sport specific strength, speed and endurance drills.  3 times per week for 6 weeks. CPL: N/D*'* | Dynamic warm-up (N/D) | wk. 1 - 2: 10 EX (LE)  wk. 3 - 4: 8 EX (LE)  wk. 5 - 6: 7 EX (LE) | None |
|  |  | None | wk. 1 - 6: 6 EX | None |
| Continued | | | | |

| *Continued* | | | | |
| --- | --- | --- | --- | --- |
| **Program**  **(Year)** | **Intervention Details** | **Dynamic warm-up** | **Plyometrics** | **Balance** |
|  |  | **Strength** | **Agility** | **Flexibility** |
| **General approach** | | | | |
| *Hölmich et al.*  *(2010)^48^* | 13 min. warm-up program to prevent groin injury. Prior to each practice (2-4 times) for one entire season. CPL: N/D | None | None | 1 EX |
|  |  | 4 EX (pair, ball, LE, core, hip/groin) | None | 1 EX (static) |
| *Hupperets et al. (2009)^23^* | 30 min. generalized proprioceptive balance board warm-up program for the prevention of ankle sprain recurrences in volleyball [1], [2] with 1-3 difficulty levels.  3 times per week for 8 weeks.  CPL: 65% | None | None | 6 EX (even surface, balance board, eyes open/ closed) |
|  |  | None | None | None |
| *Mohammadi et al. (2007)^42^* | Proprioceptive, strength, orthosis or no intervention (4 study arms) for 1 season.  CPL: N/R | N/D | N/D | Single leg stance |
|  |  | 10 sets 20 reps | N/D | N/D |
| *Nordic Hamstring*  (2006)^47^  *(2011)*^44^*  (2015)^#31^ | 10 - week progressive eccentric hamstring lowering exercise for 27 trainings sessions (into normal strength training)*. CPL: 46.8%; 91%*; 91%^#^ | None*’’ | None*’’ | None*’’ |
|  |  | 1 EX (LE, core) | None | None |
| *Waldén et al.*  *(2012)^24^* | 15 min. neuromuscular warm-up program with 4 levels and 1 partner exercise for progression targeting core stability, balance and proper knee alignment.  2 times per week for 7 months.  CPL: N/D | Jogging EX | 1 jumping EX | None |
|  |  | 5 EX (LE, core) | None | None |
| **Sports-specific approach** | | | | |
| *Cumps et al.*  *(2007)^13^* | 5 - 10 min. progressive BB specific balance training without (Pt. 1) and on balance semi globes (Pt. 2 - 4)  3 times per week (session 1 (eye open focus ball), 2 (as 1 without ball focus) and 3 (eyes closed)) over 22 weeks.  CPL: N/D | None | Pt. 1 wk. 4: 1 EX (4 variations) | Pt. 1 - 4 wk. 1 - 3, 5 - 16, 17 - 20, 21 - 22: 1EX (4 variations) |
|  |  | None | None | None |
| ACL, anterior cruciate ligament; BB, basketball; CPL, compliance; EX, exercise(s); KLIP Program, Knee Ligament Injury Prevention Program; LE, lower extremity; min., minutes; N/D, not described; OSTRC, Oslo Sports Trauma Research Center; PEP Program, Prevent injury and Enhance Performance Program; Pt., part/phase; reps, repetitions; s, seconds; UE, upper extremity; VB, volleyball; wk., week(s); yrs., years | | | | |
