# Supplementary material for: General versus sports-specific injury prevention programs in athletes: A systematic review on the effect on injury rates
Source: PLoS One. 2018 Oct 19;13(10):e0205635. doi: 10.1371/journal.pone.0205635 (PMC6195266; doi:10.1371/journal.pone.0205635)
Supplement: S2 Table — Level**: P, professional/ elite/ highest level; AM, amateur; C, coaches; cS, competitive season; CT, controlled trail; M, match/game; min., minutes; mo., months; N/D, not described; reps, repetitions; S, season; T, training/practice; wk., weeks; yrs., years. Values presented as mean ± standard deviation if not otherwise stated. (DOCX) [file pone.0205635.s002.docx]

| ***S2 Table****. Summary of characteristics of included studies for the injury rates (alphabetical order by first author)* | | | | | | | | | | |
| --- | --- | --- | --- | --- | --- | --- | --- | --- | --- | --- |
| **Author (Year) Design Country** | **Follow up**  **(mo.)** | **Population**  **(Level**)** | **Subjects** | | | | | | **Intervention** | |
|  |  |  | **Intervention** | | | **Control** | | | **Intervention** | **Control** |
|  |  |  | **N** | **Sex** | **Age in yrs.** | **N** | **Sex** | **Age in yrs.** |  |  |
| **Mixed approaches** | | | | | | | | | | |
| *Aerts et al.*  *(2013)*^21^ RCT Belgium | 6 | Basketball  (AM) | 90 | ♂: 49 ♀: 41 | ♂: 24.9 ± 4.9 ♀: 23.7 ± 5.8 | 93 | ♂: 50 ♀: 43 | ♂: 26.7 ± 5.2 ♀: 22.9 ± 3.9 | *Jump-Landing Training Program*  [3-mo./2 x wk./5-10 min.] | Followed their normal training and warm-up routine. |
| *Andersson et al.*  *(2016)*^19^ RCT (cluster) Norway |  | Handball  (P) | 331 | ♂: 171  ♀: 160 | ♂: 21.9 ± 3.7 ♀: 22.5 ± 4.2 | 329 | ♂: 168 ♀: 161 | ♂: 23.5 ± 4.8 ♀: 21.6 ± 3.3 | *OSTRC Shoulder Injury Prevention Warm-up* *Program*  [7-mo./3 x wk./10 min.] | Followed their normal warm-up routine. |
| Eils *et al.*  *(2010)^46^*  RCT (cluster)  Germany |  | Basketball  (P) | 89 | ♂: 49 ♀: 32 | 22.6 ± 6.3 | 91 | ♂: 54 ♀: 37 | 25.5 ± 7.2 | *Multistation Proprioceptive Exercise Program*  [1-S/1 x wk./20 min.] | Followed their normal warm-up routine. |
| *Emery et al.*  *(2007)*^29^  RCT (cluster)  Canada | 9 | Basketball (AM) | 494 | ♂: 244 ♀: 250 | 13 - 18 | 426 | ♂: 220 ♀: 206 | 12 - 10 | *Balance Training Program*  [18-wk./5 x wk./T/15-20 min.] | Standardized warm-up program. [10 min./T] |
| *Emery et al.*  *(2010)*^32^  RCT (cluster)  Canada |  | Soccer  (AM) | 380 | ♂: 219 ♀: 161 | 13 – 18 | 364 | ♂: 251 ♀: 113 | 13 - 18 | *Neuromuscular Prevention*  [1-yr./15 min./T] | A 15 min. warm-up program including aerobic, static and dynamic stretching. |
| *Gilchrist et al.*  *(2008)*^35^  RCT (cluster) USA |  | Soccer  (P) | 582 | ♀ | 19.88 | 852 | ♀ | 19.88 | *Prevent injury and Enhance Performance (PEP)* *Program*  [12-wk./3 x wk./< 30 min.] | Followed their normal warm-up routine. |
| Continued | | | | | | | | | | |

| *Continued* | | | | | | | | | | | | | |
| --- | --- | --- | --- | --- | --- | --- | --- | --- | --- | --- | --- | --- | --- |
| **Author (Year) Design Country** | **Follow up**  **(mo.)** | **Population (Level**)** | **Subjects** | | | | | | | | **Intervention** | | |
|  |  |  | **Intervention** | | | **Control** | | | | | **Intervention** | | **Control** |
|  |  |  | **N** | **Sex** | **Age in yrs.** | **N** | **Sex** | | | **Age in yrs.** |  |  |  |
| Hammes *et al.*  *(2014)^33^*  RCT (cluster)  Switzerland |  | Soccer (AM) | 146 | ♂ | 45 ± 8 | 119 | ♂ | | 43 ± 6 | | *FIFA 11+ Warm-up Program*  [9-mo./20 min./T] | | Followed their normal training routine. |
| *Kiani et al.*  *(2010)*^30^ CT Sweden | 9 | Soccer  (AM) | 777 | ♀ | 14.7 | 729 | ♀ | | 15.0 | | *HarmoKnee*  [9-mo./pS: 2 x wk.; cS: 1 x wk./20-25 min.] | | Followed their normal training and warm-up routine. |
| *LaBella et al.*  *(2011)^36^*  RCT (cluster)  USA |  | Soccer Basketball (AM) | 737 | ♀ | 16.2 ± 1.5 | 755 | ♀ | 16.2 ± 1.1 | | | *Neuromuscular Warm-up*  [1-S/20 min./T]  [shorten program/M] | Followed their normal warm-up routine. | |
| *Longo et al. (2012)*^37^ RCT (cluster) Italy | 9 | Basketball  (P) | 80 | ♂ | 13.5 ± 2.3 | 41 | ♂ | 15.2 ± 4.6 | | | *FIFA 11+ Warm-up Program*  [9-mo./3-4 x wk./20 min.] | Followed their normal warm-up routine. | |
| McGuine *et al.*  *(2006)^38^*  RCT  USA |  | Soccer  Basketball  (AM) | 373 | ♀: 261  ♂: 112 | 16.6 ± 1.1 | 392 | ♀: 262  ♂: 130 | | 16.4 ± 1.2 | | *Balance Training Program*  [4-wk./5 x wk. and rest of S/3 x wk.]’ | | Followed their normal conditioning routine. |
| Noyes *et al.*  *(2015)^39^*  CT  USA |  | Mixed sports  (AM) | 1000 | ♀ | 13 - 18 | 1120 | ♀ | | 13 - 18 | | *Sportsmetrics – Neuromuscular Retraining Program*  [6-wk./3 x wk./60-90 min.] | | Followed their normal training routine. |
| *Østerås et al. (2015)*^45^ RCT (pilot) Norway |  | Handball  (P) | 38 | ♀ | 15.3 ± 0.6 | 34 | ♀ | | 15.3 ± 0.7 | | *Shoulder Muscle Strength Training Program*  [8-mo./3 x wk./10-15 min.] | | No specific upper body strength training. |
| Continued | | | | | | | | | | | | | |

| Continued | | | | | | | | | | | | | | |
| --- | --- | --- | --- | --- | --- | --- | --- | --- | --- | --- | --- | --- | --- | --- |
| **Author (Year) Design Country** | **Follow up**  **(mo.)** | **Population**  **(Level**)** | | **Subjects** | | | | | | | | **Intervention** | | |
|  |  |  |  | **Intervention** | | | | **Control** | | | | **Intervention** | | **Control** |
|  |  |  |  | **N** | | **Sex** | **Age in yrs.** | **N** | **Sex** | | **Age in yrs.** |  |  |  |
| Owoeye *et al.*  *(2014)^34^*  RCT (cluster)  Nigeria |  | | Soccer (P) | 212 | | ♂ | 17.80 ± 0.94 | 204 | ♂ | | 17.49 ± 1.10 | | *FIFA 11+ Warm-up Program*  [6-mo./20 min./T] | Followed their normal warm-up routine. |
| *Pasanen et al. (2008)*^22^ RCT (cluster) Finland | 6 | Floorball  (P) | | 256 | | ♀ | 24.2 ± 5.0 | 201 | ♀ | | 23.3 ± 4.8 | | *Neuromuscular Training Program*  [6-mo./20-30 min./T] | Followed their normal training routine. |
| Silvers-Granelli *et al.*  *(2015)*^41^  RCT (cluster)  USA |  | Soccer (AM) | | 675 | | ♂ | 20.40 ± 1.66 | 815 | ♂ | | 20.68 ± 1.46 | | *FIFA 11+ Warm-up Program*  [1-S/3 x wk./15-20 min./T] | N/D |
| Silvers-Granelli *et al.*  *(2017)*^40^  RCT (cluster)  USA |  | Soccer (AM) | | 675 | | ♂ | 20 ± 2 | 815 | ♂ | | 21 ± 1 | | *FIFA 11+ Warm-up Program*  [1-S/15-20 min./T] | N/D |
| *Soligard et al.*  *(2008)*^16^  RCT (cluster)  Norway |  | Soccer (AM) | | 1055 | | ♀ | ≤16 yrs. | 837 | ♀ | | ≤16 yrs. | | *FIFA 11+ Warm-up Program*  [8-mo./20 min./T] | Followed their normal warm-up routine. |
| *Steffen et al. (2008)*^28^  RCT (cluster)  Norway |  | Soccer (AM) | | 1073 | | ♀ | 15.4 ± 0.8 | 947 | ♀ | | 15.4 ± 0.8 | | *"The 11" Prevention Program*  [8-mo./each T (15 consecutive sessions) and thereafter 1 x wk./25min.] | Followed their normal warm-up routine. |
| van Beijsterveldt *et al.*  *(2012)^43^*  RCT (cluster)  Netherlands |  | | Soccer (AM) | | 223 | ♂ | 24.4±4.1 | 233 | ♂ | 25.1±4.3 | | | *“The 11” Prevention Program*  [1-S/10-15min./T] | Followed their normal warm-up routine. |
| Continued | | | | | | | | | | | | | | |

| **Continued** | | | | | | | | | | | | | | | | | |  |
| --- | --- | --- | --- | --- | --- | --- | --- | --- | --- | --- | --- | --- | --- | --- | --- | --- | --- | --- |
| **Author (Year) Design Country** | | **Follow up**  **(mo.)** | **Population**  **(Level**)** | | | **Subjects** | | | | | | | | | **Intervention** | | |  |
|  |  |  |  |  |  | **Intervention** | | | | | **Control** | | | | **Intervention** | | **Control** |  |
|  |  |  |  |  |  | **N** | **Sex** | | **Age in yrs.** | | **N** | | **Sex** | **Age in yrs.** |  |  |  |  |
| **General approach** | | | | | | | | | | | | | | | | | |  |
| *Hölmich et al.*  *(2010)*^48^  RCT (cluster)  Denmark | |  | Soccer (AM) | | | 477 | ♂ | | 24.49 | | 430 | | ♂ | 24.62 | *Preventive Groin Warm-up Program*  [1-S/13 min./T] | | Followed their normal warm-up routine. |  |
| *Horst et al. (2015)*^31^ RCT  Netherlands | |  | Soccer  (AM) | | | 292 | ♂ | | 24.5 ± 3.6 | | 287 | | ♂ | 24.6 ± 4.1 | *Nordic Hamstring Training*  [13-wk./1-2 x wk.] | | Followed their usual training routine. |  |
| *Hupperets et al. (2009)*^23^ RCT Netherlands | | 12 | Mixed sports  (AM) | | | 256 | ♂: 136  ♀: 120 | | 28.6 ± 11.8 | | 266 | | ♂: 140  ♀: 126 | 28.0 ± 11.6 | *Proprioceptive Balance Board Training Warm-Up Program*  [8-wk./3 x wk./30 min.] | Usual care (rehabilitation) without any interference by the authors. | |  |
| *Gabbe et al. (2006)*^47^ RCT  Australia | |  | Australian Football  (P, AM) | | | 114 | ♂ | | 23.4  (18.0 - 35.0) | | 106 | | ♂ | 23.9  (17.4 - 36.0) | *Nordic Hamstring Training*  [12-wk./Club’s core training] | | Followed stretching and range of movement exercises. |  |
| *Mohammadi et al. (2007)*^42^ RCT (pilot) Iran | |  | Soccer  (P) | | | 60 | ♂ | | 24.6 ± 2.63 | | 20 | | ♂ | 24.6 ± 2.63 | *Proprioception, strength training,*  *Orthosis or no intervention*  [1-S] | | Followed usual training routine. |  |
| *Petersen et al. (2011)*^44^ RCT (cluster) Denmark | | 1 | Soccer  (P, AM) | | | 461 | ♂ | | 23.0 ± 4.0 | | 481 | | ♂ | 23.5 ± 4.0 | *Nordic Hamstring Training*  [10-wk./strength training] | Followed their normal training routine. | |  |
| *Waldén et al.*  *(2012)*^24^  RCT (cluster)  Sweden | |  | Soccer (AM) | | | 2479 | ♀ | | 14.0 ± 1.2 | | 2085 | | ♀ | 14.1 ± 1.2 | *Neuromuscular Warm-up Program*  [7-mo./2 x wk./15 min.] | Followed their normal warm-up routine. | |  |
| **Sports-specific approach** | | | | | | | | | | | | | | | | | | |
| *Cumps et al. (2007)*^13^ CT (pilot) Belgium Netherlands |  | | | Basketball (P) | 26 | | ♂: 19 ♀: 7 | ♂: 16.6 ± 1.3  ♀: 20.7 ± 7.4 | | 25 | | ♂: 16  ♀: 9 | | ♂: 17.0 ± 1.8  ♀: 19.8 ± 3.0 | *Progressive Balance Training Program*  [22-wk./3 x wk./5 -10 min.] | Followed their normal warm-up routine. | | |
| Level**: P, professional/elite/highest level; AM, amateur.  C, coaches; cS, competitive season; CT, controlled trail; M, match/game; min., minutes; mo., months; N/D, not described; reps, repetitions; S, season; T, training/practice; wk., weeks; yrs., years.  Values presented as mean ± standard deviation if not otherwise stated. | | | | | | | | | | | | | | | | | |  |
