# Supplementary material for: General versus sports-specific injury prevention programs in athletes: A systematic review on the effect on injury rates
Source: PLoS One. 2018 Oct 19;13(10):e0205635. doi: 10.1371/journal.pone.0205635 (PMC6195266; doi:10.1371/journal.pone.0205635)
Supplement: S3 Table — *B: Statistically significant difference between intervention group and baseline. ‘’: Statistically significant difference within groups. *: Statistically significant difference between intervention and control group. #: Statistically significant difference compared to all groups. A, athletes; ACL, anterior cruciate ligament; AE, athletes-exposure; aRR, adjusted rate ratio; CI, confidence interval; CG, control group; CO, compliant group; cS, competitive season; CT, controlled trail; d, days; E, exposure; EX, exercise; h, hours; IG, intervention group; INT, intervention team; IR, injury rate; IRR, adjusted incident rate ratio; LE, lower extremity; M, match, game; MD, mean difference; min., minutes; mo., months; N/A, not available; NCO, not compliant group; no., number; OR, odds ratio; pS, pre-season; RR, rate ratio; rr, relative risk; S, season; SD, standard error; T, training, practice; wk., weeks; yrs., years. Values presented as mean ± standard deviation if not otherwise stated. (DOCX) [file pone.0205635.s003.docx]

| ***S3 Table****. Results and conclusion of included studies for the injury rates (alphabetical order by first author)* | | | | |
| --- | --- | --- | --- | --- |
| **Article (year)** | **Outcome measures** | **Results** | | **Conclusion** |
|  |  | **Intervention** | **Control** |  |
| **Mixed approach** | | | | |
| *Aerts et al.*  *(2013)^21^* | **Incidence of LE injury [1000 h/E] and injury risk [HR]**  *E [h]*  **Total**  *Acute*  *Overuse* | 5226.5  N = 18; 3.6 (95% CI 1.9 – 5.25); HR 0.40 (95% CI 0.16 – 0.99)*  N = 15; 3.0 (95% CI 1.48 – 4.5); HR 0.37 (95% CI 0.12 – 1.1) N = 5; 1.0 (95% CI 0.12 – 1.87); HR 0.47 (95% CI 0.09 – 2.56) | 5009.7  N = 28; 5.4 (95% CI 3.4 – 7.3); ↑  N =21; 4.0 (95% CI 2.3 – 5.7); ↑  N =7; 1.4 (95% CI 0.35 – 2.33) | Effective prevention of LE injuries. |
| *Andersson et al. (2016)^19^* | **Prevalence of shoulder problems in the dominant arm [1-S] and injury risk [OR]**  *E [h/wk.]*  *Problems*  *Substantial* | 481  17% (95% CI 16% - 19%); OR 0.72 (95% CI 0.52 – 0.98)* 28% ↓ 5% (95% CI 4% - 6%); OR 0.78 (95% CI 0.53 – 1.16) | 505  23% (95% CI 21% - 26%); MD: 6%  8% (95% CI 7% - 9%); MD: 3% | Reduction of prevalence and risk of shoulder problems in elite handball. |
| Eils *et al.*  *(2010)^46^* | **Risk of ankle injuries [OR]**  *E [total no. sports participation]*  *Ankle injuries*  *History ankle injury*  **NNT** | 4565  N = 7; OR 0.355 (95% CI 0.151 – 0.835); p = 0.018*  OR 1.6 (95% CI 0.755– 3.553); p = 0.212  7 | 4876  N = 21 | A proprioceptive exercise program can reduce the frequency of ankle injuries as well as potential biomechanical risk factors. |
| *Emery et al.*  *(2007)^29^* | **Incidence ankle sprain [1000 player h] and injury risk [rr]**  *E [h]*  **Total**  *LE*  *Acute*  *Ankle sprain* | 39369 N = 130; 26.32 (95% CI 22.48 – 30.43)  rr 0.8 (95% CI 0.57 – 1.11) N = 106; 2.69 (95% CI 2.2 – 3.26);  rr 0.83 (95% CI 0.57 – 1.19) N = 109; 2.77 (95% CI 2.27 – 3.34);   rr 0.71 (95% CI 0.5 – 0.99)* N = 62; 1.57 (95% CI 1.21 – 2.02);   rr 0.71 (95% CI 0.45 – 1.13) | 34955 N = 141; 33.1 (95% CI 28.64 – 37.79)  N = 111; 3.18 (95% CI 2.61 – 3.82)  N = 137; 3.83 (95% CI 3.21 – 4.54)  N = 76; 2.46 (95% CI 1.97 – 3.04) | Protective effect in reducing acute-onset injuries in high school basketball. A clinically relevant trend with respect to all, lower-extremity and ankle sprain injuries. |
| *Continued* | | | | |

| *Continued* | | | | |
| --- | --- | --- | --- | --- |
| **Article (year)** | **Outcome measures** | **Results** | | **Conclusion** |
|  |  | **Intervention** | **Control** |  |
| *Emery et al.*  *(2007)^29^* | **Time lost [d]**  Mild  Moderate  Severe  **Gender effect**  *Total*  *LE*  *Acute* | 71.3% (95% CI 62.7 – 78.9)  10.1% (95% CI 5.5 – 16.6) 18.6% (95% CI 12.3 – 26.4) Injury proportion do not differ between groups.  ♀↑  rr 0.64 (95% CI 1.14 – 2.33) rr 1.64 (95% CI 1.1 – 2.38) rr 1.47 (95% CI 1.02 – 2.13) | 74.3% (95% CI 66.1 – 81.4)  16.2% (95% CI 10.4 – 23.5) 9.6% (95% CI 5.2 – 15.8) |  |
| *Emery et al.*  *(2010)^32^* | **Incidence soccer injuries [1000 player h and 100 players^1^] and injury risk [iRR]**  *E [h]*  **Total**  *Single injury*  *Two*  *Three*  *Acute onset*  *LE, ankle & knee*  *Ankle*  *History within 1 yr.*  **Severity**  *Moderate [8 – 28 d]*  *Severe [> 28 d]* | 24051  N = 50; IR 2.08 (95% CI 1.54 – 2.74) and IR 13.16 (95% CI 9.93 – 16.98)^1^; IRR 0.62 (95% CI 0.39 – 0.99); p = 0.045*; ↓38%  N = 46  N = 2  N = 42 (84% (95% CI 70.89 to 92.83)); IR 1.75 (95% CI 1.26 – 2.34); IRR 0.57 (95% CI 0.35 – 0.91); p = 0.018*; ↓43%  No difference (IR 0.126, IR 0.065 and IR 0.232, respectively); reduction trend, but not significant – point estimates  N = 14 (28% (95% CI 16.23 to 42.49)); ↑ risk in ♀ (IRR 1.86 (95% CI 0.72 – 4.8)) - point estimates  ↑ risk in ♀ (IRR 2.54 (95% CI 0.25 – 28.53) - point estimates  ↑ risk of injury for all injury definitions and players (U16–U18) and for all injury, acute-onset and ankle-sprain injury (U13–U15)  N = 13 (26% (95% CI 14.63 – 40.34))  N = 1 (2% (95% CI 0.05 – 10.64)) | 23597  N = 79; IR 3.35 (95% CI 2.65 – 4.17) and IR 21.7 (95% CI 17.58 – 26.3)^1^  N = 63  N = 5  N = 3  IR 3.05 (95% CI 2.39 – 3.84)  N = 72 (91.14% (95% CI 82.59 to 96.36))  N = 27 (34.18% (95% CI 23.81 to 45.71))  N = 13 (16.46% (95% CI 9.06 – 26.49))  N = 5 (6.33% (95% CI 2.09 – 14.16)) | Training program (on-field and home-based) has a protective effect on all injury and acute-onset injury in community youth soccer. |
| Continued | | | | |

| *Continued* | | | | | |
| --- | --- | --- | --- | --- | --- |
| **Article (year)** | **Outcome measures** | | **Results** | | **Conclusion** |
|  |  |  | **Intervention** | **Control** |  |
| *Gilchrist et al. (2008)^35^* | **Incidence ACL injury [1000 AE]**  *E [h]*  **Total knee**  *ACL*  *Noncontact*  **Training**  *Total knee*  *ACL*  *Noncontact*  **Match**  *Total knee*  *ACL*  *Noncontact*  **History of ACL**  *Total knee*  *ACL*  *Noncontact*  **Without history**  *Total knee*  *ACL*  *Noncontact* | | 26 558  N = 40; IR 1.14  N = 7; 41% ↓ IR 0.2  N = 2; 70% ↓ IR 0.06;  N = 8; IR 0.30  No ACL injury*  No ACL injury  N = 29; IR 3.37  N = 7; IR 0.81  N = 2; IR 0.23  N = 7; IR 0.2  N = 1; IR 0.03  No ACL injury*    N = 33; IR 0.94  N = 6; IR 0.17  N = 2; IR 0.06 | 41 948  N = 58; IR 1.1  N = 18; IR 0.34  N = 10; IR 0.19  N = 19; IR 0.47  N = 6; IR 0.15  N = 3; IR 0.074  N = 37; IR 2.98  N = 12; IR 0.97  N = 7; IR 0.56  N = 16; IR 0.3  N = 7; IR 0.13 N = 4; IR 0.08  N = 41; IR 0.78  N = 10; IR 0.19  N = 6; IR 0.11 | Reduction of ACL injury risk in collegiate female soccer, especially those with a history of ACL injury. |
| Hammes *et al.*  *(2014)^33^* | | **Soccer injury incidence [per 1000 h] and risk [RR]**  *Severe [n (%)]* | N = 11 (22); IR 2.6 (1.1 – 4.2); RR 0.46 (0.21 – 0.97); p = 0.04 | N = 17 (46); IR 5.8 (3 – 8.5); RR 0.46 (0.21 – 0.97); p = 0.04 | The FIFA 11+ warm-up did not yield in reductions of injury incidence in veteran soccer players. |
|  |  |  | No between-group differences in: No. of injuries (overall; training; match), Mechanism (trauma; contact; non-contact), Severity (overuse; mild; moderate), Location (upper/lower extremities, thigh, lower leg, knee, other locations, muscle/tendon, joint/ ligament, other), Reinjury (recurrence; delayed occurrence) | |  |
| *Continued* | | | | | |

| *Continued* | | | | |
| --- | --- | --- | --- | --- |
| **Article (year)** | **Outcome measures** | **Results** | | **Conclusion** |
|  |  | **Intervention** | **Control** |  |
| *Kiani et al.*  *(2010)^30^* | **Incidence any new knee injury**  **[1000 player h] and injury risk [RR]**  *E [h]*  **Total**  *Noncontact* | 66 981  N = 3; IR 0.04; 77% ↓; RR 0.23 (95% CI 0.04 – 0.83)  N = 1; IR 0.01; 90% ↓; RR 0.10 (95% CI 0.00 – 0.70)  No ACL injuries.  Injury severity ↓ | 66 505  N = 13; IR 0.20  N = 10 (5 ACL); IR 0.15 | Reduction of knee injury incidence in young female soccer. |
| *LaBella et al.*  *(2011)^36^* | **Incidence LE injury [1000 AE] and injury risk [IRR]**  *E [h]*  **Total**  *Gradual onset*  *Acute onset*  *Ankle sprain*  *Knee sprain*  *ACL sprain*  *LE surgery*  *>2 injuries*  *Gradual onset*  *Acute onset*  *Ankle sprain*  *Knee sprain*  *ACL sprain* | Intention-to-treat   28 023  N = 50; IR 1.78 (95% CI 1.29 – 2.28)* IR 0.43; 65% ↓; IRR 0.35 (95% CI 0.18 – 0.69)* IR 0.71; 56% ↓; IRR 0.71 (95% CI 0.26 – 0.76)* IR 0.25; 66% ↓; IRR 0.34 (95% CI 0.14 – 0.81)* IR 0.21; IRR 0.45 (95% CI 0.17 – 1.21) IR 0.07; IRR 0.27 (95% CI 0.06 – 1.35) IR 0; IRR N/D 3 A  Adjusted by provided personal information (855 A)  IR 0.54; IRR 0.48 (95% CI 0.18 – 1.26)  IR 0.88; IRR 0.33 (95% CI 0.17 – 0.61)* IR 0.34; IRR 0.38 (95% CI 0.15 – 0.98)* IR 0.29; IRR 0.30 (95% CI 0.10 – 0.86)* IR 0.10; IRR 0.20 (95% CI 0.04 – 0.95)* | Intention-to-treat   22 925  N = 96; IR 4.19 (95% CI 3.35 – 5.02) IR 1.22 IR 1.61 IR 0.74 IR 0.48 IR 0.26  IR 0.17* 13 A  Adjusted by provided personal information (855 A)  IR 1.12 IR 2.57  IR 0.88 IR 0.88 IR 0.46 | Reduction of noncontact LE injuries in female high school soccer and basketball. |
| Continued | | | | |

| *Continued* | | | | | |
| --- | --- | --- | --- | --- | --- |
| **Article (year)** | **Outcome measures** | **Results** | | | **Conclusion** |
|  |  | **Intervention** | **Control** | |  |
| *Longo et al. (2012)^37^* | **Incidence basketball injury [1000 AE] and injury risk [OR]**  *E [h]*  **Total**  *Training*  *Match*  *LE*  *Acute*  *Severe*  *Overuse*  *Knee*  *Ankle*  *Trunk*  *Leg*  *Hip/Groin* | 23 640  N = 14; IR 0.95 OR 0.32 (95% CI 0.17 – 0.60)*  N = 2; IR 0.14 OR 0.18 (95% CI 0.05 – 0.63)*  N = 12; IR 0.81 OR 0.48 (95% CI 0.24 – 0.97) N = 10; IR 0.68 OR 0.40 (95% CI 0.19 – 0.84)* N = 9; IR 0.61 OR 0.21 (95% CI 0.10 – 0.44)* N = 0; IR 0 OR N/D*  N = 5; IR 0.34 OR 1.21 (95% CI 0.36 – 4.11)  N = 5; IR 0.34 OR 1.21 (95% CI 0.36 – 4.11)  N = 3; IR 0.2 OR 0.79 (95% CI 0.01 – 0.72) N = 1; IR 0.07 OR 0.09 (95% CI 0.21 – 3.04)* N = 0; IR 0 OR N/D* N = 0; IR 0 OR N/D* | 12 648  N = 17; IR 2.16 N = 6; IR 0.76  N = 11; IR 1.4 N = 11; IR 1.4 N = 15; IR 1.91 N = 4; IR 0.51  N = 2; IR 0.25  N = 2; IR 0.25  N = 2; IR 0.25 N = 4; IR 0.51 N = 3; IR 0.38  N = 2; IR 0.25 | | Effective reduction of injury rates in elite male basketball. |
| McGuine *et al.*  *(2006)^38^* | **Incidence of ankle sprain [1000 AE]**  *E [AE]*  *Overall [n (%)]*  *Acute [n (%)]*  *Lateral [n (%)]*  *Medial [n (%)]*  *Syndesmotic [n (%)]*  **Ankle sprain risk**  **[n; %; rr]**  *History*  **Severity**  *Mean [d]*  *Minor [1 – 7 d]*  *Moderate [8 – 21d]*  *Severe [> 21 d]* | 20250  N = 62 (8.1); IR 1.51  N = 23 (6.1); IR 1.13  N = 56 (90.3)  N = 4 (6.4)  N = 2 (3.2)  N = 23; risk 62% (95% CI 37.8 – 101.7) of that in CG  (Kaplan-Meier survival estimate: χ^2^ = 4.00; df = 1; p = 0.045*)  rr 0.56 (95% CI 0.33 – 0.95); p = 0.033*  N = 12 (4.2); rr 2.14 (95% CI, 1.25 – 3.65); p = 0.005*;  (χ^2^ = 3.42; df = 1; p = 0.059)  7.6 (range 2 – 26); 5.8 ± 5.5  N = 40 (64.5); ↑74%  N = 18 (29); ↓22%  N = 4 (6.4) | | 20828  N = 62 (8.1); IR 1.51  N = 39 (9.9); IR 1.87  N = 39  N = 23 (7.7)  8.1 ± 6.6  59%  33% | Prevention program showed a reduction in ankle injuries by 38% in high school basketball and soccer players. |
| Continued | | | | | |

| *Continued* | | | | | |
| --- | --- | --- | --- | --- | --- |
| **Article (year)** | **Outcome measures** | **Results** | | | **Conclusion** |
|  |  | **Intervention** | | **Control** |  |
| Noyes *et al.*  *(2015)^39^* | **Noncontact ACL injury incidence rate [1000 AE]**  *E [AE]*  *Noncontact ACL* | | 36724  N = 1; IR 0.03; p = 0.03 | 61244  N = 13; IR 0.21 | Sportmetrics significantly reduced the noncontact ACL incidence rate. |
| *Østerås et al. (2015)^45^* | **Prevalence any shoulder complaints [1-S]**  *E [h/wk.]* | | 9.24 ± 3.2  ↓ (34 - 11%); Shoulder-muscle strength ↑* | 9.39 ± 2.0  ↑ (23 – 36%) | Decreases risk for shoulder complaints in handball. |
| Owoeye *et al.*  *(2014)^34^* | **Incidence [1000h] and risk [RR]**  *Total*  *Match*  *Training*  *Lower extremities*  *Acute*  *Overuse*  **Location**  **Mechanism**  **Severity**  *Mild*  *Minimal*  *Moderate*  *Severe* | | N = 36; IR 0.8; RR 0.59 (95% CI 0.40 – 0.86); p = 0.006*  N = 23; IR 7.5; RR 0.35 (95% CI 0.23 – 0.55); p < 0.001*  N = 12; IR 0.3; RR 0.93 (95% CI 0.47 – 1.86); p = 0.842  N = 26; IR 0.6; RR 0.52 (95% CI 0.34 – 0.82); p = 0.004*  N = 34; IR 0.9; RR 0.65 (95% CI 0.44 – 0.97); p = 0.037*  N = 2; IR 0.0; RR 0.26 (95% CI 0.07 – 0.98); p = 0.047*  No differences.  No differences.  N = 7; IR 0.2; RR 0.52 (0.23 – 1.21); p = 0.037*  No differences.  No differences.  No differences. | N = 94; IR 1.5  N= 73; IR 20.3  N= 22; IR 0.4  N= 76; IR 1.2  N = 80; IR 1.3  N = 14; IR 0.2  N = 27; IR 0.4 | The FIFA 11+ significantly reduced overall injury rate by 41% and lower extremity injuries by 48%. |
| Silvers-Granelli *et al.*  *(2015)*^41^ | **Injury rates [1000 AE] and risk [RR]**  **Total**  *Match [n (%)]*  *Training [n (%)]*  **Ankle [n (%)]**  **Knee [n (%)]**  *ACL [n (%)]*  **Hamstring [n (%)]**  **Time loss [d]** | | N = 285 (10.56 ± 3.64)*; IR 8.09  N = 185 (64.9) 6.85 ± 3.17*  N = 100 (35.1) 3.7 ± 2.13*  N = 59 (20.7); IR 1.675; RR 0.65 (95% CI 0.48 – 0.87)  N = 34 (11.9); IR 0.965; RR 0.42 (95% CI 0.29 – 0.61); NNT 14  N = 3 (1.1); IR 0.085; RR 0.236 (95% CI 0.193 – 0.93); NNT 70; p < 0.001*  N = 16 (5.6); IR 0.454); RR 0.37 (95% CI 0.21 – 0.63]; NNT 24; p < 0.001*  2944 (10.08 ± 14.68)* | N = 665 (19.56 ± 11.01)*; IR 15.04  N = 392 (58.9) 11.53 ± 5.84*  N = 273 (41.1) 8.03 ± 6.24*  N = 115 (17.3); IR 2.601  N = 102 (15.3%); IR 2.307  N = 16 (2.4); IR 0.362  N = 55 (8.3); IR 1.244  8790 (13.20 ± 26.6)* | The FIFA 11+ significantly reduced injury rates by 46.1% and decreased time loss by 28.6% in the competitive male collegiate soccer player. |
| Continued | | | | | |

| *Continued* | | | | |
| --- | --- | --- | --- | --- |
| **Article (year)** | **Outcome measures** | **Results** | | **Conclusion** |
|  |  | **Intervention** | **Control** |  |
| Silvers-Granelli *et al.*  *(2017)*^40^ | **Risk of ACL injury [RR] and incidence [1000 AE]**  **Total [n (%)]**  *Match [n (%)]*  *Training [n (%)]*  **Knee [n (%)]**  ***ACL***  *Contact [n (%)]*  *Non-contact [n (%)]*  *Division 1 [n (%)]*  *Division 2 [n (%)]*  Match & training  Position | N = 285 (100); IR 8.09*; RR 0.54 (95% CI 0.49 – 0.59);  p < 0.001*  N = 185 (64.9); IR 16.92; RR 0.59 (95% CI 0.52 – 0.68);  p < 0.001*  N = 100 (35.1); IR 4.01; RR 0.46 (95% CI 0.38 – 0.57);  p < 0.001*  N = 34 (11.9); IR 0.965; RR 0.42 (95% CI 0.29 – 0.61);  p < 0.001*  N = 1 (0.35); IR 0.028; RR 0.21 (95% CI 0.03 – 1.74); p = 0.148  N = 2 (0.7); IR 0.057; RR 0.25 (95% CI 0.06 – 1.15); p=0.049*  N = 2 (0.7); IR 0.114; RR 0.3 (95% CI 0.06 – 1.45); p = 0.136  N = 1 (0.35); IR 0.057; RR 0.12 (95% CI 0.02 – 0.93) p = 0.042*  No difference.  Did not affect ACL incidence. | N = 665 (100); IR 15.04  N = 392 (58.9); IR 28.77  N = 273 (41.1); IR 8.93  N = 102 (15.3); IR 2.307  N = 6 (0.9); IR 0.135  N = 10 (1.5); IR 0.226  N = 7 (1.05); IR 0.317  N = 9 (1.35); IR 0.407 | The FIFA 11+ reduces ACL injuries by 77% in competitive male collegiate soccer. |
| *Pasanen et al.*  *(2008)^22^* | **Incidence LE injury [1000 h/E] and injury risk [IRR]**  *E [h]*  **Total**  *Noncontact leg*  **Ligament**  *Ankle*  *Knee*  **Muscle strain** | 32327 N = 87; 66% ↓; IRR 0.70 (95% CI 0.52 – 0.93)*  N = 20; IR 0.65 (95% CI 0.37 – 1.13)*;  IRR 0.34 (95% CI 0.20 – 0.57)*  N = 15; IR 0.48 (95% CI 0.27 – 0.84);  IRR 0.35 (95% CI 0.19 – 0.64)*  N = 8; IR 0.27 (95% CI 0.11 – 0.64);  IRR 0.28 (95% CI 0.12 – 0.67)*  N = 7; IR 0.22 (95% CI 0.10 – 0.45);  IRR 0.49 (95% CI 0.18 – 1.31)  N = 5; IR 0.15 (95% CI 0.06 – 0.37);  IRR 0.40 (95% CI 0.12 – 1.32) | 25019 N = 102 N = 52; IR 2.08 (95% CI 1.58 – 2.72)  N = 38; IR 1.52 (95% CI 0.16 – 0.60)  N = 27; IR 1.10 (95% CI 0.71 – 1.70)  N = 11; IR 0.44 (95% CI 0.24 – 0.80)  N = 14; IR 0.57 (95% CI 0.29 – 1.12) | Program is effective in preventing acute non-contact injuries of the legs in female floorball players. |
| Continued | | | | |

| *Continued* | | | | |
| --- | --- | --- | --- | --- |
| **Article (year)** | **Outcome measures** | **Results** | | **Conclusion** |
|  |  | **Intervention** | **Control** |  |
| *Soligard et al.*  *(2008)^16^* | **Incidence LE injury**  **[1000 player h] and injury risk [RR]**  E [h]  **Total**  *Match*  *Training*  *Overuse*  *Severe*  **>2 injuries**  **Re-injury**  **Total**  *Match*  *Training*  *Overuse*  *Severe*  *Acute*  *Severe*  *Contact*  *Severe*  **Body category**  *Knee*  **Acute**  *Contusion*  **Overuse**  *Tendon pain*  *Lower back pain* | 3.9 ± 0.2 (SD) (8.1 ± 0.5 (SD) (M); 1.9 ± 0.2 (SD) (T)); RR 0.71 (95% CI 0.49 – 1.03) | | The risk of injury can be reduced by about one third and the risk of severe injuries by as much as a half in young female footballers. |
|  |  | Intention-to-treat  49899 (16 057 (M); 33 842 (T)) N = 161; ↓; RR 0.68 (95% CI 0.48 – 0.98)*  No difference. No difference.  ↓; RR 0.47 (95% CI 0.26 – 0.85)* ↓; RR 0.55 (95% CI 0.36 – 0.83)*  No difference; RR 0.46 (95% CI 0.20 – 1.01)  Per protocol  N = 161; ↓; RR 0.68 (95% CI 0.56 – 0.84)*  ↓; RR 0.71 (95% CI 0.55 – 0.91)*  ↓; RR 0.63 (95% CI 0.44 – 0.90)*  ↓; RR 0.44 (95% CI 0.27 – 0.71)*  ↓; RR 0.30 (95% CI 0.14 – 0.68)*  ↓; RR 0.76 (95% CI 0.61 – 0.95)*  ↓; RR 0.65 (95% CI 0.43 – 0.97)*  ↓; RR 0.64 (95% CI 0.45 – 0.90)*  ↓; RR 0.54 (95% CI 0.38 – 0.78)*  0.7 ± 0.1 (SD); RR 0.55 (95% CI 0.36 – 0.84)*  0.3 ± 0.1 (SD); RR 0.44 (95% CI 0.24 – 0.80)*  0.2 ± 0.1 (SD); RR 0.48 (95% CI 0.23 – 0.99)*  0.0 ± 0.0 (SD); RR 0.11 (95% CI 0.01 – 0.91)* | 45428 (14 342 (M); 31 086 (T))  N = 215  ↑; RR 0.51 (95% CI 0.29 – 0.87)*  1.3 ± 0.2 (SD)  0.7 ± 0.1 (SD)  0.5 ± 0.1 (SD)  0.2 ± 0.0 (SD) |  |
| *Steffen et al. (2008)^28^* | **Overall incidence [1000 h/E] and injury risk [RR]**  E [h]  Overall  Acute  E [h]  Overall  Acute | Intention-to-treat    66423 (20 731 (M); 45 692 (T)) IR 3.6 (95% CI 3.2 – 4.1); RR 1.0 (95% CI 0.8 – 1.2) IR3.2 (95% CI 2.7 – 3.6); RR 1.0 (95% CI 0.8 – 1.2) No difference in type, location or severity compared with CG.  CO (per protocol; 14 teams) 19 093 (5 371 (M); 13 722 (T)) IR 3.4 (95% CI 2.5 – 4.2); RR 0.9 (95% CI 0.7 – 1.2) IR 2.8 (95% CI 2.0 – 3.5); RR 0.9 (95% CI 0.6 – 1.2) No difference with NCO and CG. | 65725 (19 856 (M); 45 869 (T)) IR 3.7 (95% CI 3.2–4.1) IR 3.2 (95% CI 2.8–3.6)  NCO (per protocol; 44 teams) 47330 (15 360 (M); 31 970 (T)) IR 3.8 (95% CI 3.2 –4.3); RR 0.9 (95% CI 0.7 – 1.2) IR 3.3 (95% CI 2.8 – 3.9); RR 0.8 (95% CI 0.6 – 1.1) | No effect of the injury prevention program on the injury rate. |
| *Continued* | | | | |

| *Continued* | | | | |
| --- | --- | --- | --- | --- |
| **Article (year)** | **Outcome measures** | **Results** | | **Conclusion** |
|  |  | **Intervention** | **Control** |  |
| van Beijsterveldt *et al.*  *(2012)^43^* | **Injury incidence**  **[per sports h]**  *E [median, IQR]*  **Total [n (% players)]**  *Match [%]*  *Training [%]*  **Injury severity** *[%]*  *Slight (0 d)*  *Minimal (1 – 3d)*  *Mild (4 – 7d)*  *Moderate (8 – 28d)*  *Severe (> 28d)*  *Career Ending*  *D off [median, IQR]*  **Injury Mechanism**  *Acute [%]*  *Overuse [%]*  **Recurrent Injury [%]**  **Injury location [%]**  Ankle  Upper leg posterior  Knee  Groin  Upper leg anterior  Other | 103.4; IR 31.4  N = 207 (60.5); IR 9.6 (95% CI 8.4 – 11)  65.4; IR 21.1 (95% CI 17.8 – 25)  34.6; IR 3.7 (95% CI 2.8 – 4.8)  0  5.9  18.5  46.3  28.8  0.5  14, 28.5  78.9  21.1  13  21.8  18.4  11.7*  9.7  8.3  30.1 | 104.3; IR 35  N = 220 (59.7); IR 9.7 (95% CI 8.5 – 11.1)  69.6; IR 22.7 (95% CI 19.3 – 26.7)  30.4; IR 3.1 (95% CI 2.3 – 4)  0.5  5.1  21.5  41.6  29.9  1.4  17, 30  82.7  17.3  14.1  16.3  13.4  19.8*  11.4  12.9  26.2 | The 11 did not find significant differences in overall injury incidence or injury severity between intervention and control group. |
| **General approach** | | | | |
| *Hölmich et al.*  *(2010)^48^* | **Time to 1^st^ groin injury [HR]**  *E [h]*  *Intervention*  *Pre-injury*  *Level of play* | N/D  31% ↓; HR 0.69 (95% CI 0.40 – 1.19) N = 183; HR 1.97* N = 369 (low level); HR 2.58* | N/D  N = 203 N = 300 (low level) | No effect of the intervention.  Pre-injury doubles and higher level of play almost triples the risk of a new groin injury. |
| Continued | | | | |

| *Continued* | | | | | | |  |
| --- | --- | --- | --- | --- | --- | --- | --- |
| **Article (year)** | **Outcome measures** | **Results** | | | | **Conclusion** |  |
|  |  | **Intervention** | | | **Control** |  |  |
| *Horst et al. (2006)^31^* | **Incidence hamstring injury [1000 player h] and risk [OR]**  **E [H]**  *Total*  *Match*  *Training*  **Total [n (%)]**  *Overall*  *Match*  *Training*  *Position*  **Severity [d]**  *Slight (0 d)*  *Minimal (1 – 3 d)*  *Mild (4 – 7 d)*  *Moderate (8 – 28 d)*  *Severe (> 28 d)* | 90.5 ± 5.4  34.0 ± 13.8  56.5 ± 17.0  31 ± 15  N = 11 (31); OR 0.628 (95% CI, 0.197 – 1.999); p = 0.427; in follow up N = 6; rr 3.384 (95% CI 1.362 – 8.409); OR 0.282 (95% CI 0.110 – 0.721); p = 0.005*  IR 0.7 (95% CI 0.6 – 0.8)  IR 1.2 (95% CI 0.82 – 1.94)  IR 0.33 (95% CI 0.25 – 0.46)  No differences.  N = 4  N = 2 | | | 96.6 ±16.0  35.1 ± 14.3  61.5 ± 17.7  28 ± 19  N = 25 (69); in follow up N = 18  IR 0.7 (95% CI 0.6 – 0.8)  IR 1.2 (95% CI 0.82 – 1.94)  IR 0.33 (95% CI 0.25 – 0.46)  N = 1  N = 1  N = 2  N = 5  N = 9 | Incorporating the Nordic hamstring exercise in regular amateur training significantly reduces hamstring injury incidence, but it does not reduce hamstring injury severity. |  |
| *Gabbe et al. (2006)*^47^ | **Risk hamstring injury [rr]**  **Hamstring [%]** | | 4; rr 1.2, 95% CI 0.5- 2.8  Only protective effect when athletes participate in the first two sessions (rr 0.3, 95% CI 0.1-1.4; p=0.098) | 13.2 | | Eccentric exercise helped reducing hamstring injuries. However, poor compliance makes it difficult to assess. | |
| *Mohammadi et al.*  *(2007)*^42^ | **Incidence [1000 AE] and risk [rr] of ankle sprains**  *Proprioception*  *Strength*  *Orthosis* | | N = 1; rr 0.13 (95% CI 0.003 – 0.93); p = 0.02*  N = 4; rr 0.50 (95% CI 0.11 – 1.87); p = 0.27  N = 2, rr 0.25 (95% CI 0.03 – 1.25); p = 0.06 | N = 8; rr 0.13 (95% CI 0.003 – 0.93) | | Proprioception training reduced ankle sprain rates. | |
| *Hupperets et al. (2009)^23^* | **Incidence recurrent ankle sprain**  **[1000 h/E] and injury risk [rr]**  *E [h]*  *Overall*  **Time lost incidence**  **[1000 h/E]** | | 30140  N = 56 (22%); 35% ↓;  IR 1.86 (95% CI 1.37 – 2.34); rr 0.63 (95% CI 0.45 – 0.88)*  Recurrence ↓ in not medically treated compared to counterparts*  IR 0.65 (95% CI 0.38 – 0.92); rr 0.53 (95% CI 0.32 – 0.88)* | 30682  N = 89 (33%);  IR 2.90 (95% CI 2.30 – 3.50)  IR 1.17 (95% CI 0.82 – 1.52) | | Effective for preventing self reported recurrent ankle sprains (specifically beneficial if not medically treated). | |
| *Continued* | | | | | | |  |

| *Continued* | | | | |
| --- | --- | --- | --- | --- |
| **Article (year)** | **Outcome measures** | **Results** | | **Conclusion** |
|  |  | **Intervention** | **Control** |  |
| *Petersen et al. (2011)^44^* | **Incidence hamstring injury [100 player S] and injury risk [aRR]**  *E [h]*  **Total [no.]**  *Overall*  *New*  *Recurrent*  ***Time lost [d]*** | N/D  N = 12 new; N = 3 recurrent; pS: N = 9 (60%)  No injuries while performing the Nordic EX.  IR 3.8; aRR 0.293 (95% CI 0.150 – 0.572)*  IR 3.1; aRR 0.410 (95% CI 0.180 – 0.933)*  IR 7.1; aRR 0.137 (95% CI 0.037 – 0.509)* 454 (N = 15) | N/D  N = 32 new; N = 20 recurrent; pS: N = 12 (23%)  ↑; aRR 1.76 (95% CI 0.54-5.67) IR 13.1  IR 8.1  IR 45.8  1344 (N = 52) | Additional eccentric hamstring EX can decrease the rate of overall, new, and recurrent acute hamstring injuries. |
| *Waldén et al.*  *(2012)^24^* | **Incidence knee injury [1000 h/E] and injury risk [RR]**  *E [h]* **Total**  **ACL**  Noncontact  Severe  Any acute  **ACL**  Noncontact  Severe  Any acute | Intention-to-treat  149214 N = 49  N = 7 A (0.28%); 64% ↓ RR 0.36 (95% CI 0.15 – 0.85)*  RR 0.40 (95% CI 0.13 – 1.18) N = 26 A (1.05%); RR 0.70 (95% CI 0.42 – 1.18) N = 48 A (1.94%); RR 0.92 (95% CI 0.61 – 1.40)  CO (1303 A)  83% ↓; RR 0.17 (95% CI 0.05 – 0.57)*  RR 0.26 (95% CI 0.07 – 0.99)*  RR 0.18 (95% CI 0.07 – 0.45)*  RR 0.53 (95% CI 0.30 – 0.94)* | Intention-to-treat  129084  N = 47 N = 14 A (0.67%)  N = 31 A (1.49%)  N = 44 A (2.11%)  CO (1967 A) | Reduction of ACL injury rate in adolescent female football players. |
| *Continued* | | | | |

| *Continued* | | | | |
| --- | --- | --- | --- | --- |
| **Article (year)** | **Outcome measures** | **Results** | | **Conclusion** |
|  |  | **Intervention** | **Control** |  |
| **Sports-specific approach** | | | | |
| *Cumps et al.  (2007)^13^* | **Incidence ankle sprain [1000 h/E] and injury risk [rr]**  *E [h]*  *Total E*  *Basketball E*  **New injury**  *Total E*  *Basketball E*  **Re-injury**  *Total E*  *Basketball E*  **Gender effect**  ♀  ♂ | N/D  IR 1.19 (95% CI 0.15 – 2.25); rr 0.34 (95% CI 0.12 – 0.96)* IR 1.22 (95% CI 0.15 – 2.29); rr 0.30 (95% CI 0.11 – 0.84)*  N/D; rr 0.21 (95% CI 0.03 – 1.44) IR 0.78 (95% CI 0.10 – 1.76); rr 0.15 (95% CI 0.02 – 1.15)  IR 0.44 (95% CI 0.42 – 1.30); rr 0.76 (95% CI 0.17 – 3.40) IR 0.36 (95% CI 0.34 – 1.05); rr 0.44 (95% CI 0.10 – 1.91)  IR 0.83 (95% CI 0.79 – 2.44); rr 0.30 (95% CI 0.03 – 2.87) IR 1.39 (95% CI 0.03 – 2.75); rr 0.29 (95% CI 0.09 – 0.93)* | N/D  IR 3.54 (95% CI 1.23 – 5.85)  IR 4.09 (95% CI 1.42 – 6.76)  N/D IR 1.03 (95% CI 0.03 – 2.03)  IR 3.00 (95% CI 0.37 – 5.63) IR 1.72 (95% CI 0.21 – 3.22)  IR 2.76 (95% CI 1.06 – 6.58) IR 4.74 (95% CI 1.23 – 8.26) | The use of balance training is recommended as a routine during basketball activities for the prevention of ankle sprains. |
| *^B^: Statistically significant difference between intervention group and baseline.  ‘’ : Statistically significant difference within groups.  * : Statistically significant difference between intervention and control group.  ^#^ : Statistically significant difference compared to all groups.  A, athletes ; ACL, anterior cruciate ligament; AE, athletes-exposure; aRR, adjusted rate ratio; CI, confidence interval; CG, control group; CO, compliant group; cS, competitive season; CT, controlled trail; d, days; E, exposure; EX, exercise; h, hours; IG, intervention group; INT, intervention team; IR, injury rate; IRR, adjusted incident rate ratio; LE, lower extremity; M, match, game; MD, mean difference; min., minutes; mo., months; N/A, not available; NCO, not compliant group; no., number; OR, odds ratio; pS, pre-season; RR, rate ratio; rr, relative risk; S, season; SD, standard error; T, training, practice; wk., weeks; yrs., years.  Values presented as mean ± standard deviation if not otherwise stated. | | | | |
